# Retraction: The Tumor Suppressor Role of miR-124 in Osteosarcoma

**DOI:** 10.1371/journal.pone.0214018

**Published:** 2019-03-13

**Authors:** Shuo Geng, Xiaojun Zhang, Jie Chen, Xing Liu, Hepeng Zhang, Xiaoyan Xu, Yan Ma, Baoxin Li, Yunqi Zhang, Zhenggang Bi, Chenglin Yang, and The PLOS ONE Editors

After publication of this article [1] concerns were raised about results reported in Figs 3 and 5:

- There are similarities between the 24h panels of MG-63 cells and U2OS cells in Fig 3A.
- In Fig 3B, there are similarities between the "untreated" and "scramble" panels for both the MG-63 and U2OS cells, as well as a repeated section in the "miR-124 inhibitor" panels for both the MG-63 and U2OS cells.
- In Fig 3C, the "untreated" panel for the MG-63 cells is repeated in the "scramble" panel for U2OS cells. There is also a repeated section in the "miR-124 mimic" panels for both cells.
- In Fig 5C, there is a repeated section in the panels labeled "Rac1" and "Rac1+miR-124."

The authors noted that the original data underlying Figs 3 and 5 are no longer available. The authors requested retraction due to concerns about the reliability of the data reported in the article.

In light of the above concerns, the authors and *PLOS ONE* Editors retract this article.

The authors agreed with the retraction.
